# Effects of recreational small-sided games from different team sports on the improvement of aerobic fitness in youth sedentary populations: A systematic review

**DOI:** 10.1016/j.heliyon.2023.e22041

**Published:** 2023-11-14

**Authors:** Tingyu Li, Qi Xu, Shuang Wang, Kai Qi, Peng Su, Rui Miguel Silva, Hugo Sarmento, Filipe Manuel Clemente

**Affiliations:** a Research Center in Sports Performance, Recreation, Innovation and Technology (SPRINT), 4960-320, Melgaço, Portugal; b Gdansk University of Physical Education and Sport, 80-336, Gdańsk, Poland; c Changsha Xiangjun Peicui Expermental Middle School, Changsha, 410002, China; d Chizhou Third People's Hospital, Chizhou, 247100, China; e Escola Superior Desporto e Lazer, Instituto Politécnico de Viana Do Castelo, Rua Escola Industrial e Comercial de Nun’Álvares, 4900-347, Viana Do Castelo, Portugal; f University of Coimbra, Research Unit for Sport and Physical Activity (CIDAF), Faculty of Sport Sciences and Physical Education, Coimbra, Portugal

**Keywords:** Exercise, Human physical conditioning, Health promotion, Healthy lifestyle, Physical fitness

## Abstract

Aerobic fitness is a critical aspect of overall health and well-being, essential for maintaining a high quality of life. Unfortunately, sedentary behavior has been on the rise among young adults, and this has had a negative impact on their aerobic fitness levels. Therefore, it is crucial to identify enjoyable physical exercise training programs that can play a pivotal role in improving aerobic fitness. The objective of this study was to systematically review the experimental studies concerning the impact of small-sided games (SSGs) training programs, implemented across various team sports, on the enhancement of aerobic fitness in a youth sedentary population. A literature search was conducted in PubMed, Scopus, and the Web of Science on August 01, 2023. Our eligibility criteria focused on studies involving sedentary youth populations (aged <18 years) as the target population. These studies needed to incorporate interventions based on SSGs as the intervention of interest, comparing them to passive or control groups as the comparator. The primary outcomes of interest were related to maximal oxygen uptake (VO2max), assessed either directly or indirectly, or the results of field-based cardiorespiratory tests. We specifically considered two- or multi-arm randomized controlled studies as the study design of interest. Out of the initial pool of 1980 studies, we reviewed 38 full-text articles, ultimately selecting and analyzing 13 studies for inclusion in our review. Among the studies included, a total of 1281 participants were enrolled in SSG-based interventions, while 744 participants acted as part of the control groups. Regarding the impact on VO2max, the six studies that investigated this outcome showed varying improvements, ranging from 2.2 % to 31.3 % when participants were exposed to SSGs. In terms of the outcome related to endurance performance in field-based tests, the eight studies that examined this aspect found that participants exposed to SSGs showed improvements ranging from 0.1 % to 79.8 %. In conclusion, this systematic review suggests that SSG-based interventions conducted among sedentary youth populations can play a positive role in improving their aerobic fitness. This improvement in aerobic fitness can have potential positive impacts on their overall health and quality of life.

## 1 Introduction

Aerobic fitness, encompassing critical aspects such as maximal oxygen uptake (VO2max) and endurance performance [1], plays a pivotal role in promoting health and overall well-being through a variety of mechanisms [2]. Among these mechanisms, one of the most prominent is its favorable impact on the cardiovascular system. This includes improvements in blood circulation, a lowered resting heart rate, and a reduced workload on the heart, thereby mitigating the risk of heart-related ailments [3].

Moreover, aerobic fitness further augments the body's ability to optimize oxygen utilization [4]. As individuals engage in regular aerobic exercise, their muscles become more proficient at extracting and utilizing oxygen from the bloodstream to generate energy [5]. This adaptation is pivotal for the improvement of exercise performance.

Thus, being physically active and improving aerobic fitness can be pivotal for ensuring a healthier and more well-being population [6,7]. To promote aerobic fitness on a broader scale, it is essential to offer diverse training programs to the general population. For instance, prolonged-duration, low-to-moderate intensity aerobic exercises have been shown to have positive effects on the aerobic fitness of sedentary individuals [8]. However, high-intensity interval training (HIIT), characterized by short yet highly intense bouts of exercise, has proven to be of paramount importance and highly effective for enhancing aerobic fitness [[9], [10], [11]].

Among the various forms of HIIT, incorporating drill-based games can be particularly appealing to individuals who enjoy team sports. Engaging in recreational sports participation, such as participating in recreational small-sided games (SSGs) practices, may provide greater motivation and enjoyment for individuals [12]. By offering diverse and engaging exercise options, it becomes more feasible to encourage sedentary individuals to incorporate physical activity into their daily routines, effectively reducing their health risks.

SSGs represent widely utilized training methodologies in team sports, serving the dual purpose of enhancing both physiological and physical demands, as well as technical and tactical skills simultaneously [13,14]. SSGs involve the alteration of the standard playing format of a team sport, making it smaller and more constrained [15].

During SSGs, players engage in various sport-specific movements. These games, due to their smaller participant numbers, also require more intense physical effort, leading to increased oxygen uptake and higher energy expenditure [16,17]. Consequently, SSGs can make a substantial contribution to improvements in aerobic fitness [45]. While targeting a sedentary population, there is extensive research examining the capacity of these individuals to engage in activities that are naturally tailored to their initial fitness levels [[18], [19], [20], [21], [22], [23], [24], [25]]. Despite their sedentary status, participants demonstrate an ability to become increasingly involved in the games, intensifying their physical activity levels as the games progress. These findings are so compelling that various systematic reviews have robustly described the benefits for sedentary individuals across age groups, ranging from youth to older adults [[18], [19], [20], [21], [22], [23], [24], [25]].

It is noteworthy that existing literature includes diverse systematic reviews focused on recreational small-sided games in football, exploring various outcomes such as bone health, fat mass, and maximal oxygen uptake [[18], [19], [20], [21], [22], [23], [24], [25]]. However, it is important to highlight that SSGs are not limited to soccer; other sports such as basketball, handball, and rugby have also been employed as recreational small-sided games [[26], [27], [28]].

Given the substantial and mounting body of results affirming the effectiveness of recreational SSGs across various sports [[26], [27], [28]], it has become imperative to undertake systematic reviews to gain a comprehensive understanding of their impact on enhancing the aerobic fitness of sedentary populations. A meticulous review, systematically discerning the characteristics of these training programs and their effects on diverse markers of aerobic fitness, holds the potential to offer valuable insights. Such insights can serve to bolster community interventions and guide policies aimed at augmenting physical activity levels among sedentary individuals.

This systematic review distinguishes itself from others by summarizing the key findings of experimental studies across various recreational team sports, deviating from the existing reviews that primarily focus on football. Consequently, our systematic review is uniquely poised to explore the diverse formats of SSGs offered in team sports, including basketball, soccer, handball, rugby, and others. Its principal aim is to summarize the primary outcomes related to aerobic fitness identified in studies involving sedentary youth populations utilizing SSGs. This review will exclusively include randomized two- or multi-arm study designs. By delving deeply into the available body of literature, this review aspires to furnish evidence-based recommendations for the integration of SSGs into physical activity regimens tailored for sedentary youth.

## 2 Methods

### 2.1 The registration

This systematic review meticulously followed the PRISMA 2020 guidelines [29], which are widely acknowledged as the gold standard for reporting systematic reviews. Our review's protocol was diligently registered in advance on the OSF platform, bearing the code number DOI 10.17605/OSF.IO/NT9BU (accessed on August 19, 2023).

### 2.2 Eligibility criteria

We included all original articles published in peer-reviewed journals, as well as those with ahead-of-print status, in this systematic review. Language restrictions were not applied to the articles [30]. We adhered to the PICOS approach to establish our eligibility criteria, which are detailed in Table 1. In brief, our criteria centered on studies involving sedentary youth populations (aged <18 years) as the target demographic. These studies were required to feature interventions based on SSGs as the primary intervention, with comparisons made to passive or control groups as the comparator. Our primary outcomes of interest included measures related to VO2max, assessed either directly or indirectly, or the results of field-based cardiorespiratory tests. We specifically focused on two- or multi-arm randomized controlled study designs.

**Table 1:** Eligibility criteria.

	Inclusion Criteria	Exclusion Criteria
Population	The study encompassed youth populations (aged <18 years old) with no restrictions on sex or clinical conditions. Participants were categorized into two tiers based on the participant classification framework [31]: Tier 0, representing those engaged in sedentary behavior, and Tier 1, representing recreationally active individuals. Specifically, Tier 0 participants did not meet the minimum activity guidelines and could be considered inactive, while Tier 1 participants met the World Health Organization's minimum activity target and/or engaged in multiple sports or forms of physical activity.	The review explicitly excludes adults (>18 years old) and youths enrolled in Tiers 2 to 5 of the participant classification framework [53]. This means that individuals beyond the age of 18 and those classified as Tier 2 to Tier 5 participants, which may indicate higher levels of physical activity or specific athletic profiles, were not included in the study's scope. The focus of the review remains on youth populations within Tier 0 (sedentary behavior) and Tier 1 (recreationally active) of the classification framework.
Intervention	The review encompassed participants who were exposed to structured small-sided games training programs, without imposing any limitations on the duration or frequency of the training programs. Moreover, there were no constraints on the training volume or intensity, allowing for a comprehensive analysis of the effects of such training on the participants.	The review excluded participants who were exposed to training programs based on sports other than small-sided games (SSGs) in the context of team sports. Additionally, participants who were exposed to a combination of small-sided games and other training interventions were not considered within the scope of this review.
Comparator	The review included both passive control groups, which were not exposed to any other training interventions and maintained their regular physical activity levels and lifestyle, as well as active control groups. The active control groups were exposed to other exercise programs; however, these programs did not involve small-sided games training.	The review excluded participants who were already exposed to training programs incorporating small-sided games. The primary objective was to assess the effects of structured small-sided games training programs in comparison to other control conditions, making sure to exclude any potential overlap with participants who were already engaged in similar small-sided games training programs.
Outcomes	The review included assessments of aerobic fitness outcomes, such as maximal oxygen uptake, maximal aerobic speed, and anaerobic threshold.	The review excluded studies that focused on acute physiological and/or physical responses, which typically pertain to the immediate reactions during a single training session or exercises. Furthermore, the review did not consider any other physical fitness markers not directly related to aerobic fitness, such as strength, power, speed, change of direction, flexibility, mobility, or body composition.
Study design	The review included studies with randomized controlled multi-arm designs	The review excluded studies that did not utilize randomized designs or controlled designs.

The relevance of articles was assessed using the PICOS (Table 1) approach to examine the comprehensive content and establish the criteria for inclusion and exclusion. For those that required a more thorough evaluation, the full texts were carefully scrutinized to determine their suitability for inclusion in the review.

### 2.3 Data sources

The process of identifying relevant studies involved searching various databases, including: (i) PubMed, (ii) Scopus, (iii) SPORTDiscus, and (iv) Web of Science, on August 01, 2023. To ensure no pertinent materials were overlooked, manual searches were also conducted on the reference lists of the included studies.

### 2.4 Search strategy

The search was systematically conducted using Boolean operators AND/OR, with no filters or restrictions applied to the date of publication or language. This approach was chosen to maximize the chances of identifying relevant studies [32]. The comprehensive search strategy is outlined in Table 2 found below.

**Table 2:** Full search strategy for each database.

Database	Specificities of the databases	Search Strategy	Number of articles
PubMed	Search for title and abstract also includes keywords	(("small-sided games" [Title/Abstract] OR "sided-games" [Title/Abstract] OR "drill-based games" [Title/Abstract] OR "SSG" [Title/Abstract] OR "conditioned games" [Title/Abstract] OR "small-sided and conditioned games" [Title/Abstract] OR "reduced games" [Title/Abstract] OR "play formats" [Title/Abstract] OR "recreational*" [Title/Abstract]) AND ("team sport" [Title/Abstract] OR football [Title/Abstract] OR soccer [Title/Abstract] OR futsal [Title/Abstract] OR handball [Title/Abstract] OR volleyball [Title/Abstract] OR basketball [Title/Abstract] OR hockey [Title/Abstract] OR rugby [Title/Abstract] OR cricket [Title/Abstract] OR "water polo" [Title/Abstract] OR lacrosse [Title/Abstract] OR softball [Title/Abstract] OR korfball [Title/Abstract])) AND (aerobic*[Title/Abstract] OR endurance*[Title/Abstract] OR "cardiorespiratory" [Title/Abstract] OR "maximal oxygen uptake" [Title/Abstract] OR "maximal aerobic speed" [Title/Abstract] OR "locomotor profile" [Title/Abstract] OR "distance covered" [Title/Abstract] OR "ventilatory threshold" [Title/Abstract] OR "running performance" [Title/Abstract] OR fitness [Title/Abstract])	376
Scopus	Search for title and abstract also includes keywords	(TITLE-ABS-KEY ("small-sided games" OR "sided-games" OR "drill-based games" OR "SSG" OR "conditioned games" OR "small-sided and conditioned games" OR "reduced games" OR "play formats" OR "recreational*") AND TITLE-ABS-KEY ("team sport" OR football OR soccer OR futsal OR handball OR volleyball OR basketball OR hockey OR rugby OR cricket OR "water polo" OR lacrosse OR softball OR korfball) AND TITLE-ABS-KEY (aerobic* OR endurance* OR "cardiorespiratory" OR "maximal oxygen uptake" OR "maximal aerobic speed" OR "locomotor profile" OR "distance covered" OR "ventilatory threshold" OR "running performance" OR fitness))	706
SPORTDiscus	Search for title and abstract also includes keywords	AB (“small-sided games” OR “sided-games” OR “drill-based games” OR “SSG” OR “conditioned games” OR “small-sided and conditioned games” OR “reduced games” OR “play formats” OR “recreational*”) AND AB (“team sport” OR football OR soccer OR futsal OR handball OR volleyball OR basketball OR hockey OR rugby OR cricket OR “water polo” OR lacrosse OR softball OR korfball) AND AB (aerobic* OR endurance* OR "cardiorespiratory" OR "maximal oxygen uptake" OR "maximal aerobic speed" OR "locomotor profile" OR "distance covered" OR "ventilatory threshold" OR "running performance" OR fitness)	283
Web of Science	Search for title and abstract also includes keywords and its designated “All Fields”	“small-sided games” OR “sided-games” OR “drill-based games” OR “SSG” OR “conditioned games” OR “small-sided and conditioned games” OR “reduced games” OR “play formats” OR “recreational*” (Abstract) AND “team sport” OR football OR soccer OR futsal OR handball OR volleyball OR basketball OR hockey OR rugby OR cricket OR “water polo” OR lacrosse OR softball OR korfball (Abstract) AND aerobic* OR endurance* OR "cardiorespiratory" OR "maximal oxygen uptake" OR "maximal aerobic speed" OR "locomotor profile" OR "distance covered" OR "ventilatory threshold" OR "running performance" OR fitness (All Fields)	615

### 2.5 Selection process

The screening process was meticulously conducted by two authors (TL and HS), who independently reviewed the retrieved records, including titles and abstracts. Subsequently, they individually assessed the full texts of the selected records. In cases where discrepancies arose, a collaborative reevaluation was undertaken to reach a consensus. If a consensus could not be reached, a third author (FMC) was responsible for making the final decision. We utilized EndNote X9.3.3 software, developed by Clarivate Analytics in Philadelphia, PA, USA, to manage the records.

### 2.6 Data extraction process

The data collection process was carried out independently by the authors TL and FMC. In instances where disagreements arose, RMS assumed the role of an arbiter to resolve any discrepancies. To streamline and maintain organization throughout this process, we created a dedicated Microsoft® Excel datasheet. This datasheet encompassed all relevant data and key information, facilitating a structured and efficient approach to data management.

### 2.7 Outcome measures

We collected data pertaining to participants and contextual information, including variables such as the date of publication, primary research objectives, sample size, country of origin, age, gender distribution, clinical information, and competitive level.

Regarding intervention-related details, we documented information about the timing of the competitive season, program duration, training frequency, adherence to the training regimen, and various dose-related aspects, including duration, repetitions, rest intervals, intensity, frequency, and training density. Additionally, we recorded specifics about the rules of play, the format of play, and pitch size.

Comparators were also documented, encompassing information about passive control groups or active control groups, along with details about the type of exercise, exercise intensity, and volume.

Our primary focus in collecting outcome measures was on endurance performance. This included variables such as (i) directly or indirectly measured VO2max, (ii) directly or indirectly measured maximal aerobic speed (MAS), (iii) distance covered in a field-based endurance test, (iv) time to exhaustion in a test, and (v) ventilatory threshold, among other relevant measures.

### 2.8 Study risk of bias assessment

To assess the risk of bias in the included studies, we employed the Physiotherapy Evidence Database (PEDro) scale [33]. This scale has undergone previous validation and demonstrated reliability. It evaluates eleven specific items, with ten of them contributing to the overall score assigned to each article. Scores range from 0, indicating the lowest quality, to 10, representing the highest quality. Typically, cut-off scores are used to categorize articles into qualitative categories, such as 'poor' (<4 points), 'fair' (4–5 points), 'good' (6–8 points), and 'excellent' (9–10 points).

The assessment process involved two authors independently reviewing and rating the articles using the PEDro scale. Following this, the two authors (XX and XX) compared their scores and engaged in detailed discussions to address any discrepancies item by item. In cases where a consensus could not be reached, a third author (XX) was consulted, providing their score and ultimately making the final decision regarding the rating.

## 3 Results

### 3.1 Study identification and selection

**Fig. 1:**
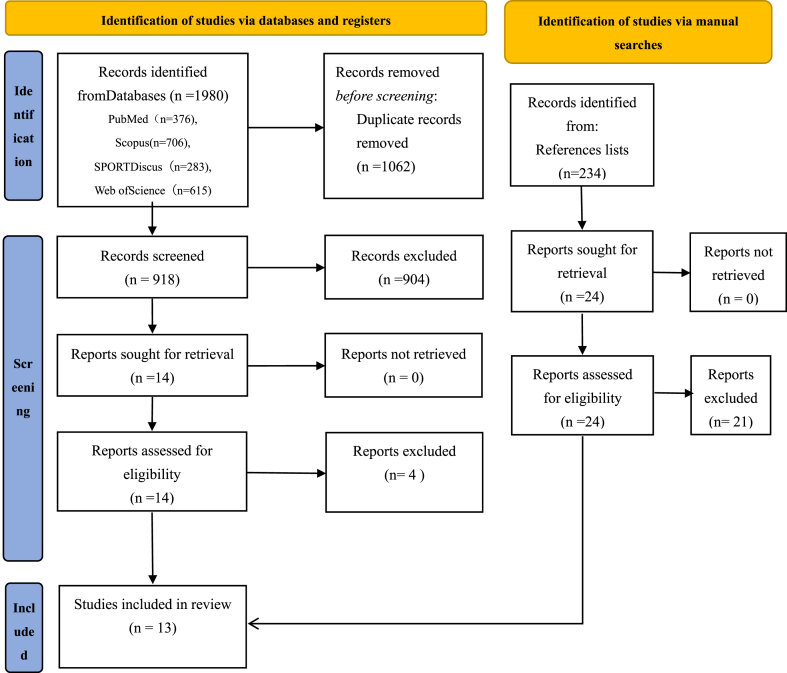
PRISMA flow diagram.

The initial search yielded a total of 1980 studies, as depicted in Fig. 1. After removing duplicated entries (1062 studies) through automated and manual processes, 918 unique studies remained. These were subjected to further evaluation for relevance, considering both titles and abstracts. Subsequently, 904 studies were excluded. A thorough review of the full texts was then conducted for the remaining 14 studies. Following this review, four studies [[34], [35], [36], [37]] were excluded from the analysis as they did not meet the predetermined inclusion criteria, specifically because their research did not focus on aerobic fitness. Finally, 10 eligible studies were selected.

To ensure the precision of the outcomes, we conducted a comprehensive cross-referencing of the literature. Among the four systematic reviews or meta-analyses [18,23,38,39], a meticulous re-evaluation of 234 relevant sources was carried out. Ultimately, this rigorous cross-referencing procedure identified three studies that met the criteria for inclusion.

Concurrently, when combined with the previously identified 10 studies, a total of 13 studies were conclusively determined to be suitable for inclusion.

### 3.2 Assessment of the risk of bias

Within the scope of this systematic review, we analyzed a collection of thirteen studies (Table 3). Among these, three studies demonstrated a quality score ranging from 4 to 5 points, indicating a moderate level of quality. In contrast, the remaining ten studies received a quality score ranging from 6 to 8 points, signifying a notably elevated level of quality.

**Table 3:** Physiotherapy Evidence Database (PEDro) scale ratings.

Study	C1	C2	C3	C4	C5	C6	C7	C8	C9	C10	C11	Score
[40]	0	1	0	1	0	0	0	1	1	1	1	6
[41]	0	1	0	1	0	0	0	1	1	1	1	6
[42]	0	1	0	1	0	0	0	1	1	1	1	6
[43]	0	1	0	1	0	0	0	1	1	1	1	6
[44]	1	1	0	1	1	1	0	1	1	1	1	8
[45]	0	1	0	0	0	0	0	1	0	1	1	4
[46]	0	1	0	1	0	0	0	1	1	1	1	6
[47]	0	1	0	1	0	0	0	1	0	1	1	5
[48]	1	1	0	1	0	1	0	1	1	1	1	7
[49]	0	1	0	1	0	0	0	1	1	1	1	6
[50]	1	1	0	0	0	1	0	1	1	1	1	6
[51]	0	1	0	0	0	0	0	1	1	1	1	5
[52]	0	1	0	1	0	0	0	1	1	1	1	6

C1: eligibility criteria were specified; C2: subjects were randomly allocated to groups; C3: allocation was concealed; C4: the groups were similar at baseline regarding the most important prognostic indicators; C5: there was blinding of all subjects; C6: there was blinding of all therapists who administered the therapy; C7: there was blinding of all assessors who measured at least one key outcome; C8: measures of at least one key outcome were obtained from more than 85 % of the subjects initially allocated to groups; C9: all subjects for whom outcome measures were available received the treatment or control condition as allocated, or, where this was not the case, data for at least one key outcome were analyzed according to “intention to treat”; C10: the results of between-group statistical comparisons are reported for at least one key outcome; C11: the study provides both point measures and measures of variability for at least one key outcome.

It is worth noting that a major deficiency observed across all articles was related to the lack of reported information on blinding procedures for subjects, investigators, and evaluators. Unfortunately, none of the studies included in the analysis employed a protocol that ensured concealment of allocation throughout the investigation.

### 3.3 Characteristics of the included studies

Table 4 provides a comprehensive overview of the key characteristics of the studies included in this review. Notably, all conducted experiments adhered to a randomized controlled group design. The SSGs training interventions involved a total of 1281 participants, while the control groups consisted of 744 individuals. Among the compiled studies, eight studies [[40], [41], [42], [43],^45^,^47^,^49^,^50^] exclusively focused on male participants, while one study [46] centered solely on female participants. Furthermore, four studies [44,48,51,52] included both genders in their study reports.

**Table 4:** Characteristics of the included studies.

Study	Clinical Registration	Country	N	Age (Years)	Sex	Assessmnents (Number)	Tests Applied	Outcomes Presented in the Study
[40]	IN191046	Saudi Arabia	24	19.60 ± 0.96	Boys	3 (pre, mid,post)	stage treadmill walking test	VO2max measurements
[41]	Not reported	NA	22	15.9 ± 0.6	Boys	3 (pre, mid,post)	YYIRT	YYIRT distance
[42]	Not reported	NA	41	15.9 ± 0.6	Boys	2 (pre, post)	YYIRT	YYIRT distance
[43]	Not reported	China	40	9 to 10	Boys	3 (pre, mid,post)	20-m shuttle run test	VO2max measurements
[44]	NCT02000492	Denmark	239	8 to 10	Both	2 (pre, post)	YYIR1C	YYIR1Csubmax (% of HRmax); YYIR1C performance
[45]	Not reported	NA	42	11 to 13	Boys	2 (pre, post)	Yo-Yo Intermittent Endurance test	Yo-Yo test distance; HRmax; RHR
[46]	Not reported	NA	59	13.3 ± 0.3	Girls	2 (pre, post)	YYIRT	YYIRT distance
[47]	REC-010712	NA	53	17.0 ± 0.6	Boys	2 (pre, post)	multistage fitness test	VO2max measurements
[48]	TCTR20150512001	Brazil	20	14.1 ± 1.3	Both	2 (pre, mid,post)	Cardio-respiratory fitness test	VO2max measurements; HR peak
[49]	Not reported	NA	22	19.63 ± 0.67	Boys	3 (pre, mid,post)	stage treadmill walking test	VO2max measurements
[50]	NCT02000492	NA	291	11 to 13	Boys	2 (pre, post)	YYIR1C	YYIR1C performance
[51]	H-16026885	Denmark	1122	10 to 12	Both	2 (pre, post)	YYIR1C	YYIR1C performance; VO2max measurements
[52]	Not reported	NA	105	15.7 ± 0.6	Both	2 (pre, post)	YYIRT	YYIRT distance

NA: not available; VO2max:maximal oxygen uptake; HR: heart rate; HRmax:maximal heart rate; RHR:resting heart rate; YYIRT: yo-yo intermittent recovery test level 1; YYIR1C:Yo-Yo Intermittent Recovery Level 1 Children’s Test.

Table 5 offers a detailed breakdown of the SSG-based training regimens utilized in the included studies. Notably, the most common training duration ranged from 8 weeks to 10 months, with session durations varying from a minimum of 12 min to a maximum of 60 min. Regarding training frequency, the majority of studies implemented 3 sessions per week, although this ranged from a minimum of 2 to a maximum of 5 sessions per week. The SSG formats predominantly used were 3v3, with a prevailing preference for an area of 40–70 m^2^ per player.

**Table 5:** Characteristics of SSG-based programs in the included studies.

Study	Training Attendance	Duration	Days Per Week	Total Sessions	Training Duration (min)	SSG formas	SSG pitch dimension (length × width)	SSG area per player (m^2^)	specific training	Sets (n)	Recovery (min)	Work Duration (min)	Work Intensity	Training Drills
[40]	100 %	12 weeks	2	24	30	NA	40 × 30 m	NA	handball	NA	NA	NA	87.8%HRmax	warm up (10 min); SSGs (10 min); cool-down (10 min)
[41]	60–69 %	6 months	3	54	60	4v4 to 7v7	20 × 15 m to 60 × 30 m	NA	soccer	NA	NA	NA	80 ± 8 % HRmax	NA
[42]	NA	8 weeks	2	16	NA	4v4 to 6v6	30mx20 m to 50mx30 m	NA	soccer	NA	NA	NA	NA	Warm-up; SSGs (30–45 min)
[43]	>95 %	10 weeks	3	30	60	NA	NA	NA	soccer	NA	NA	NA	NA	Warm-up (10 min); SSGs (40 min); cool-down (10 min)
[44]	NA	10months	5	NA	12	3v3	NA	NA	soccer; basketbal; unihockey	NA	NA	NA	73.9 ± 5.3 % HRmax	NA
[45]	NA	12 weeks	3	36	60	5v5 to 7v7	NA	80 m^2^	soccer	NA	NA	NA	84.5 ± 4.1 % HRmax	Warm-up (10 min); SSGs (40 min); cool-down (10 min)
[46]	NA	12 weeks	2	24	40	NA	NA	NA	soccer; basketball; handball; volleyball	NA	NA	NA	NA	Warm-up (10 %); SSGs (80 %); cool-down (10 %)
[47]	NA	8 weeks	NA	28	60	NA	NA	NA	soccer	NA	NA	NA	NA	NA
[48]	>90 %	12 weeks	3	36	60	2v2; 3v3; 4v4	NA	NA	soccer	NA	NA	NA	NA	Warm-up (10 min); SSGs (40 min); cool-down (10 min)
[49]	91.66 %	12 weeks	2	24	30	3v3	NA	NA	basketball	NA	NA	NA	NA	SSGs (30 min)
[50]	>50 %	10months	NA	NA	60	3v3; 4v4	20 × 13 m	NA	soccer; basketball; floorball; team handball or volleyball	NA	NA	NA	75.1 ± 2.3 % HRmax	SSGs (40min)
[51]	NA	11 weeks	NA	2	NA	NA	NA	NA	soccer	NA	NA	NA	NA	SSGs (45min)
[52]	>85 %	8 months	NA	NA	NA	3v3; 4v4	NA	40–70 m^2^	soccer	4	3	5	NA	SSGs (45min)

SSGs: small-sided games; NA: not available; HRmax: maximal heart rate.

Table 6 provides a detailed overview of the characteristics of the control groups in the included studies. Out of these, three groups adopted a passive role, while 10 groups actively engaged in various forms of exercise. Among the active groups, the most commonly performed exercises included high-intensity interval training and mandatory physical education activities.

**Table 6:** Characteristics of control groups.

Study	Characteristic	Duration/Frequency	Attendance	Training Intensity	Training Drills
[40]	One control group	12 weeks/2 session	NA	87.8%HRmax	NA
[41]	a control group who maintained their normal level of physical activity	6 months/thrice a week	NA	NA	Kept their regular physical activity level
[42]	Control (inactive)	NA	NA	NA	Kept their regular physical activity level
[43]	a control group who maintained their normal level of physical activity	10 weeks/NA	NA	NA	Kept their regular physical activity level
[44]	One Interval running group and one control group	10months/NA	NA	NA	NA
[45]	One high-intensity interval training group and one control group	12 weeks/NA	NA	NA	NA
[46]	a control group that participated only in compulsory physical education	NA	NA	NA	Kept their regular physical activity level
[47]	Control (inactive)	NA	NA	NA	Kept their regular physical activity level
[48]	Control (inactive)	12 weeks/NA	NA	NA	NA
[49]	One control group	12 weeks/NA	NA	NA	NA
[50]	a control group who maintained their normal level of physical activity	10 months/NA	NA	NA	Kept their regular physical activity level
[51]	a control group that participated only in compulsory physical education	11 weeks/2 sessions week	NA	NA	Kept their regular physical activity level
[52]	a control group that participated only in compulsory physical education	8 months/2 sessions week	NA	NA	Kept their regular physical activity level

NA: not available; HRmax: maximal heart rate.

### 3.4 Results of individual studies

Table 7 illustrates significant differences in VO2max results before and after the intervention, comparing the SSG group to the control group. Out of the six studies [40,43,[47], [48], [49],^51^] that examined the impact of SSGs versus control groups on VO2max, five studies [43,[47], [48], [49],^51^]reported significant improvements in participants' VO2max as a result of SSGs. Additionally, the between-group comparisons (i.e., SSGs vs. control) showed significant differences after the intervention in two studies [47,48]. Among the studies that demonstrated significant improvements of VO2max within the SSG groups, the duration of the intervention varied between 8 and 12 weeks.

**Table 7:** Results of maximal oxygen uptake before and after the intervention.

Study	Group	Outcome	Baseline	Post-intervention	% dif (Post-Pre)/Pre*100	Within group comparison*	Between group comparison*
[40]	SSG	VO2max	34.53 ± 2.49 ml/kg/min	35.28 ± 2.87 ml/kg/min	2.2	Not significant	Not significant (p = 0.129)
	Control	VO2max	36.96 ± 3.60 ml/kg/min	36.38 ± 3.51 ml/kg/min	−1.6	Not significant	Not significant (p = 0.129)
[43]	SSG	VO2max	46.8 ± 1.8 ml/kg/min	48.4 ± 2.0 ml/kg/min	3.4	Significantly improved (p < 0.05; ES: 0.84)	Significantly different (p = 0.037)
	Control	VO2max	46.0 ± 2.6 ml/kg/min	46.3 ± 2.1 ml/kg/min	0.7	Not significant	Significantly different (p = 0.037)
[47]	SSG	VO2max	52.1 ± 4.6 ml/kg/min	52.9 ± 4.5 ml/kg/min	1.5	Significantly improved (p < 0.05)	Significantly different (p = 0.012)
	Control	VO2max	48.8 ± 4.6 ml/kg/min	50.1 ± 3.8 ml/kg/min	2.7	Not significant	Significantly different (p = 0.012)
[48]	SSG	VO2max	25.2 ± 3.2 ml/kg/min	33.1 ± 9.2 ml/kg/min	31.3	Significantly improved (p < 0.001)	Significantly different (p = 0.013)
	Control	VO2max	22.9 ± 3.1 ml/kg/min	24.0 ± 3.9 ml/kg/min	4.8	Not significant (p = 0.237)	Significantly different (p = 0.013)
[49]	SSG	VO2max	34.54 ± 2.93 ml/kg/min	35.66 ± 3.06 ml/kg/min	3.2	Not described	Significantly different (p = 0.046)
	Control	VO2max	37.10 ± 4.31 ml/kg/min	36.17 ± 3.54 ml/kg/min	−2.5	Not described	Significantly different (p = 0.046)
[51]	SSG	VO2max	14.31 ± 1.64 ml/kg/min	14.87 ± 2.01 ml/kg/min	3.9	Not described	Significantly different (p = 0.02)
	Control	VO2max	14.76 ± 1.74 ml/kg/min	14.74 ± 1.86 ml/kg/min	−0.1	Not described	Significantly different (p = 0.02)

VO2max:maximal oxygen uptake; * inferential results reported by the individual studies; p: p-value; ES: effect size.

Table 8 displays the results of the Yo-Yo test performance for both the SSG group and the control group before and after the intervention. Out of the eight studies [41,42,[44], [45], [46],[50], [51], [52]] that examined the impact of SSGs versus control groups on endurance performance, six studies [41,42,44,46,50,52] reported significant improvements in participants' endurance performance as a result of SSGs. Additionally, the between-group comparisons (i.e., SSGs vs. control) showed significant differences after the intervention in one studies [45]. Among the studies that demonstrated significant improvements of endurance performance within the SSG groups, the duration of the intervention varied between 8 weeks and 10 months.

**Table 8:** Results of endurance performance after intervention.

Study	Group	Outcome	Baseline	Post-intervention	% dif (Post-Pre)/Pre*100	Within group comparison	Between group comparison
[41]	SSG	YYIRT1 (m)	650 ± 129	851 ± 199	30.9	Significantly improved (p = 0.005; ES = 1.5)	Not described
	Control	YYIRT1 (m)	685 ± 153	834 ± 182	21.8	Significantly improved (p = 0.03; ES = 0.90)	Not described
[42]	SSG	YYIRT1 (m)	1042 ± 249	NA	27.5	Significantly improved (>95–99.5 % likely)	Significantly different (ES = 1.82)
	Control	YYIRT1 (m)	662 ± 157	NA	30.9	Significantly improved (>75–95 % likely)	Significantly different (ES = 1.82)
[44]	SSG	YYIR1C (m)	682 ± 450	845 ± 490	23.9	Not described	Not significant
	HIIT	YYIR1C (m)	647 ± 450	820 ± 480	26.7	Not described	Not significant
	Control	YYIR1C (m)	712 ± 411	838 ± 447	17.7	Not described	Not significant
[45]	SSG	YYIRT1 (m)	476.0 ± 182.4	856.0 ± 456.0	79.8	Significantly improved (p < 0.05)	Not significant (p = 0.057)
	HIIT	YYIRT1 (m)	567.2 ± 305.6	1028.0 ± 552.4	81.2	Significantly improved (p < 0.05)	Not significant (p = 0.057)
	Control	YYIRT1 (m)	722.8 ± 576.0	857.2 ± 542.0	18.6	Not significant	Not significant (p = 0.057)
[46]	SSG	YYIRT1 (m)	964.7 ± 242.3	1066.7 ± 265.9	10.6	Significantly improved (ES = 0.40)	Significantly different (p = 0.004)
	Control	YYIRT1 (m)	947.6 ± 286.2	994.5 ± 290.9	4.9	Significantly improved (ES = 0.16)	Significantly different (p = 0.004)
[50]	SSG	YYIR1C (m)	759 ± 463	909 ± 490	19.8	Not described	Not significant
	Control	YYIR1C (m)	719 ± 413	847 ± 448	17.8	Not described	Not significant
[51]	SSG	YYIR1C (m)	535.55 ± 340.42	660.68 ± 447.91	23.4	Not described	Significantly different (p = 0.02)
	Control	YYIR1C (m)	613.22 ± 401.14	620.41 ± 419.92	1.2	Not described	Significantly different (p = 0.02)
[52]	SSG	YYIRT1 (m)	1504.4 ± 93.1	1537.8 ± 95.1	2.2	Significantly improved (p < 0.05)	Not significant (p = 0.072)
	Control	YYIRT1 (m)	1487.1 ± 99.2	1488.2 ± 96.8	0.1	Not significant	Not significant (p = 0.072)

NA: not available; YYIRT: yo-yo intermittent recovery test level 1; YYIR1C:Yo-Yo Intermittent Recovery Level 1 Children’s Test; HIIT: running-based high-intensity interval training.

## 4 Discussion

Our systematic review sought to provide a quantitative summary of the influence of SSGs-based training programs on the VO2max and endurance performance of sedentary youth populations. In summary, among the thirteen studies included, our findings demonstrate that SSG-based interventions are consistently and significantly effective in enhancing the aerobic fitness (both VO2max and endurance performance) of sedentary individuals when implemented for a minimum of eight weeks. Furthermore, in comparison to control groups, SSGs generally outperformed passive control groups in significantly improving aerobic fitness, while demonstrating similar improvements to running-based high-intensity interval training approaches.

### 4.1 Exploring SSG-induced VO2max adaptations in sedentary youth populations

The summary of individual studies indicated that SSG-based intervention programs conducted on sedentary youth populations showed a significant trend toward improving aerobic fitness when compared to control groups [40,43,[47], [48], [49],^52^]. Although no specific systematic review was dedicated to sedentary youth populations, our results align with a previous systematic review in recreational soccer [53]. This previous review [53], focused on adults, found that recreational soccer produced substantial improvements in VO2max compared to strength training or passive control groups. Additionally, it highlighted that VO2max improvements were greater with SSGs compared to moderate-continuous aerobic exercise.

Significant enhancements in VO2max were confirmed in individual studies included in our review. For instance, the individual studies [[47], [48], [49],^51^] conducted using soccer and basketball SSGs demonstrated significant improvements in VO2max after the intervention. Our systematic review has shed light on the significant effectiveness of SSGs when applied over an 11 to 12-week duration, featuring player formats ranging from 2v2 to 4v4 [[47], [48], [49],^51^]. Notably, these formats are renowned for their high intensity, resulting in heart rate responses that typically range from 75 % HRmax to 85 % [45,48]. Interestingly, among adults, these values can even reach 90 % HRmax for more than 20 % of the time [[54], [54a],^55^]. Additionally, the rate of perceived exertion during SSGs tends to be lower than during continuous running and much lower than during interval training. This aspect holds substantial importance for increasing adherence to long-term exercise programs, as the lower perceived stress can help individuals achieve their cardiorespiratory adaptation goals more comfortably.

Moreover, SSGs offer a unique advantage from a psychological perspective. They do not lead to a high resistance to training fatigue and are often more enjoyable than other exercise programs, such as running-based approaches. Furthermore, SSGs foster a greater degree of social interaction compared to running groups [56], which can enhance the overall exercise experience and contribute to long-term adherence.

Nevertheless, it is imperative to acknowledge that our investigation predominantly focused on male youth, with sporadic instances of gender-mixed participation. Particularly conspicuous is the dearth of comprehensive inquiry into the singular impact of aerobic fitness among female adolescents. Furthermore, upon reflection, it is discernible that soccer has emerged as the preeminent team sport scrutinized, thus underscoring the paucity of research encompassing other sporting disciplines [40,49]. Hence, prospective inquiries should contemplate the integration of a broader spectrum of team sports, thereby unveiling the quintessential SSG interventions most efficacious in engendering augmented aerobic fitness within leisurely and sedentary youth populations.

### 4.2 Exploring SSG-induced endurance performance adaptations in sedentary youth populations

Our systematic review also delved into analyzing the impact of SSGs-based interventions on endurance performance, primarily assessed through field-based tests. The Yo-Yo Intermittent Recovery test was the most commonly employed assessment in these studies. Upon summarizing the findings from individual studies, it became evident that within-group improvements were consistently significant in the groups exposed to SSG-based interventions [41,42,[44], [45], [46],[50], [51], [52]]. Furthermore, when compared to passive control groups, our analysis revealed that SSG-based interventions were significantly more effective in enhancing endurance performance [51,52]. However, in comparison to active control groups (such as running-based high-intensity interval training), the differences were not statistically significant [44,45]. It is worth noting that both SSGs and active control groups showed significant improvements in the included studies [44,45].

Our findings align with previous reviews conducted in middle-aged and older adult populations, which also found improvements in endurance performance after exposing sedentary adults to SSG-based interventions [57]. Our results also align with a systematic review that summarized the effects of recreational soccer on endurance performance in pediatric populations [58].

Endurance performance in tests like the Yo-Yo Intermittent Recovery test depends not only on improvements in the aerobic system but also on factors related to change-of-direction economy and the ability to recover between bouts [59]. SSGs typically consist of high-intensity efforts performed repeatedly during the game, interspaced with periods of lower effort. It is expected that this format can positively impact participants' ability to recover between bouts and become more efficient during intense efforts. This improvement may be due, among other factors, to increased mitochondrial content and oxygen consumption in muscle tissue, leading to improved oxygen extraction from capillaries [60,61]. Additionally, SSGs require higher levels of neural drive during sprints and activate anaerobic glycolysis, recruiting additional fast motor units for short bursts of intense effort [62].

The combined effect of engaging in high-intensity efforts with demands reaching maximal physical exertion can be particularly appealing for youth populations. Simultaneously, it ensures that they enjoy the games and choose the sport that suits them best to stay active [63]. Therefore, when implementing SSGs, it is crucial to ensure a high level of individual participation. To achieve this, practitioners may opt for smaller formats of play (2v2 to 4v4) and larger pitch sizes (e.g., 75–100 m^2^). This approach aims to maximize individual efforts while promoting maximal technical involvement in these games [64].

### 4.3 Limitations and future research trends

The current literature on SSGs has significantly enhanced our understanding of their effects, particularly on aerobic fitness. However, there are notable limitations that deserve attention and further investigation.

First, we need a more comprehensive exploration of SSGs' impact on various physiological and psychological dimensions beyond just aerobic fitness. While existing studies have highlighted improvements in aerobic endurance, other physiological markers remain underexplored. Examining psychological aspects like motivation, enjoyment, and mental well-being could provide a more holistic perspective on SSGs' influence.

Second, the limited diversity in participant demographics is a current research constraint. Most studies have focused on sedentary adolescents, limiting generalizability. To better understand SSG interventions, we should explore their efficacy across diverse populations with varying fitness levels and cultural backgrounds.

Third, the temporal dynamics of SSG benefits require further investigation. Many studies have used short intervention periods (typically 8–12 weeks), potentially missing long-term adaptations and sustainability. Longer-term studies with follow-up assessments could shed light on the durability of improvements in aerobic fitness and sprint speed.

Fourth, the assessment of the risk of bias reveals a consistent absence of blinding procedures in relation to participant assessment and intervention. In forthcoming studies, it is imperative to implement these blinding procedures rigorously to mitigate potential biases arising from the intervention or assessment processes.

Lastly, this systematic review encompassed a range of individual studies, which examined pre-pubescent [43, 44, 45, 50], post-pubescent [41, 42, 46, 47, 48, 52], and young adult participants (>18 years old, [40, 49]). Therefore, it is essential to approach the summary of these individual studies with caution, taking into account the potential influence of biological maturation factors that could account for variations in the results. However, it is noteworthy that despite these different developmental stages, the individual studies generally indicated positive effects in the groups concerning improvements in the primary outcomes.

In the future, SSG research should adopt a multifaceted approach, incorporating advanced physiological assessments, thorough psychosocial analyses, and a more diverse participant pool. Longitudinal studies extending beyond typical intervention durations, along with follow-up assessments, can reveal sustained SSG impacts. Collaborations across disciplines and the integration of wearable technology offer promising avenues for understanding mechanisms and optimizing SSG interventions.

## 5 Conclusions

This systematic review is centered on assessing the advantages of recreational SSGs for enhancing aerobic fitness among sedentary adolescents who engage in team sports. The study findings underscore the notable impact of these games on enhancing VO2max in leisurely and sedentary young individuals when compared to other control groups. Furthermore, both the HIIT and the SSGs intervention groups exhibited substantial enhancements in endurance performance, as evidenced by the results of the Yo-Yo intermittent recovery test. These outcomes underscore the effectiveness of SSGs training programs in augmenting aerobic fitness among recreational, sedentary youth populations.

However, it is essential to acknowledge a limitation: the majority of the studies focused on football, which limits the diversity needed to comprehend the impact on other sports fully. Furthermore, most of the studies had relatively short durations, typically ranging from 8 to 12 weeks, which doesn't allow us to fully grasp the long-term effects of these strategies. To address this limitation, future studies should consider increasing sample sizes, diversifying the sports used, and extending the study periods to longitudinal studies lasting months or even years to gain a more comprehensive understanding of the impact into adulthood.

## Financial support

Nothing to declare.

## CRediT authorship contribution statement

**Tingyu Li:** Writing – review & editing, Writing – original draft, Methodology, Investigation. **Qi Xu:** Writing – review & editing, Writing – original draft. **Shuang Wang:** Writing – review & editing, Writing – original draft. **Kai Qi:** Writing – review & editing, Writing – original draft. **Peng Su:** Writing – review & editing, Writing – original draft. **Rui Miguel Silva:** Writing – review & editing, Writing – original draft. **Hugo Sarmento:** Writing – review & editing, Writing – original draft. **Filipe Manuel Clemente:** Writing – review & editing, Writing – original draft, Supervision, Methodology, Conceptualization.

## Declaration of competing interest

The authors declare that they have no known competing financial interests or personal relationships that could have appeared to influence the work reported in this paper.
